# Mannose-modified hyaluronic acid nanocapsules for the targeting of tumor-associated macrophages

**DOI:** 10.1007/s13346-022-01265-9

**Published:** 2022-12-06

**Authors:** Iago Fernández-Mariño, Clément Anfray, Jose Crecente-Campo, Akihiro Maeda, Aldo Ummarino, Carmen Teijeiro-Valiño, Dario Blanco-Martinez, Francis Mpambani, Laurence Poul, Julie Devalliere, Matthieu Germain, Juan Correa, Marcos Fernandez-Villamarin, Paola Allavena, Eduardo Fernandez-Megia, María José Alonso, Fernando Torres Andón

**Affiliations:** 1 Center for Research in Molecular Medicine and Chronic Diseases (CiMUS), Campus Vida, Universidade de Santiago de Compostela, Santiago de Compostela, 15782 Spain; 2 Department of Pharmacology, Pharmacy and Pharmaceutical Technology, Campus Vida, Universidade de Santiago de Compostela, Santiago de Compostela, 15782 Spain; 3 Health Research Institute of Santiago de Compostela (IDIS), Santiago de Compostela, 15706 Spain; 4 Laboratory of Cellular Immunology, IRCCS Humanitas Research Hospital, Rozzano-Milan, 20072 Italy; 5 Nanomag Laboratory, Applied Physics Department, Campus Vida, Universidade de Santiago de Compostela, Santiago de Compostela, 15782 Spain; 6 Curadigm 60 rue de Wattignies, Paris, 75012 France; 7 Departamento de Química Orgánica, Centro Singular de Investigación en Química Biolóxica e Materiais Moleculares (CIQUS), Universidade de Santiago de Compostela, Jenaro de la Fuente s/n, Santiago de Compostela, 15782 Spain

**Keywords:** Cancer, Hyaluronic acid, Mannose, Polymeric nanocapsules, Nanoprimer, Tumor-associated macrophages

## Abstract

Tumor-associated macrophages (TAMs), a class of immune cells that play a key role in tumor immunosuppression, are recognized as important targets to improve cancer prognosis and treatment. Consequently, the engineering of drug delivery nanocarriers that can reach TAMs has acquired special relevance. This work describes the development and biological evaluation of a panel of hyaluronic acid (HA) nanocapsules (NCs), with different compositions and prepared by different techniques, designed to target macrophages. The results showed that plain HA NCs did not significantly influence the polarization of M0 and M2-like macrophages towards an M1-like pro-inflammatory phenotype; however, the chemical functionalization of HA with mannose (HA-Man) led to a significant increase of NCs uptake by M2 macrophages in vitro and to an improved biodistribution in a MN/MNCA1 fibrosarcoma mouse model with high infiltration of TAMs. These functionalized HA-Man NCs showed a higher accumulation in the tumor compared to non-modified HA NCs. Finally, the pre-administration of the liposomal liver occupying agent Nanoprimer™ further increased the accumulation of the HA-Man NCs in the tumor. This work highlights the promise shown by the HA-Man NCs to target TAMs and thus provides new options for the development of nanomedicine and immunotherapy-based cancer treatments.

**Graphical abstract:**

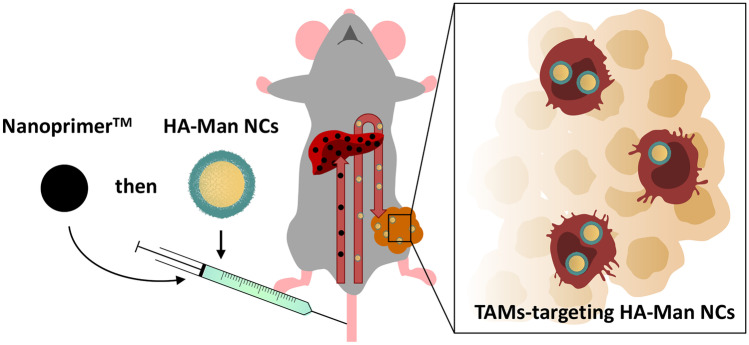

**Supplementary Information:**

The online version contains supplementary material available at 10.1007/s13346-022-01265-9.

## Introduction

Tumor-associated macrophages (TAMs) play a major role in tumor progression [1]. They exhibit a chronic-inflammatory phenotype (M2-like) that promotes immunosuppression in the tumor microenvironment through the production of chronic inflammatory cytokines, interleukins, prostaglandins, and transforming growth factors [2, 3]. TAMs also modulate other aspects of tumor progression, such as angiogenesis, cell proliferation, fibrosis, and metastasis [4]. Therefore, these immunosuppressive cells are a relevant target for cancer prognosis and treatment [1, 4].

In parallel, nanotechnological approaches stand out for their versatility to design a large number of nanostructures adapted to different therapeutic objectives, including TAMs targeting [5, 6]. In this context, lipid nanoparticles [7, 8], liposomes [9–12], and polymeric nanoparticles [13, 14], among other nanocarriers, have been designed to reach TAMs for prognosis purposes or as therapies to kill them, impair their protumoral functions, or reprogram them towards M1-like macrophages with antitumoral activity [6, 15].

Hyaluronic acid (HA) is an endogenous glycosaminoglycan, involved in the architectural structure of tissues, which has been used as a polymer to design TAM-targeted nanocarriers [16–18]. HA is recognized by several receptors, among them the CD44 receptor, which is widespread in cancer, endothelial, and immune cells [19, 20]. Several studies have demonstrated that the HA molecular weight, HA/receptor ratio, and/or receptor isoforms could limit its targeting capacity [21]. With the aim of exploiting the passive targeting capacity of nanostructures, our research group has developed different types of HA-based nanocarriers [22–26], particularly nanoparticles (NPs) and nanocapsules (NCs). NCs are versatile systems that have the ability to harbor in their oily core different types of drugs, such as small hydrophobic molecules, but also larger macromolecules, either in their outer polymeric layer or in their interphase. Previously, we have dedicated intensive efforts to design HA NCs capable of delivering anticancer drugs to the tumor microenvironment [27]. More precisely, we have developed docetaxel-loaded HA NCs that show an important antitumoral efficacy in a lung cancer murine model [28]. We have also invested significant effort in adapting HA-based nanotechnologies for the delivery of polynucleotides [29, 30], peptides [31], proteins [23, 32], and monoclonal antibodies [33], in the context of cancer. Nevertheless, the HA targeting capacity has shown mixed results, probably due to the abovementioned widespread presence of the CD44 receptor in different types of cells. Thus, we have decided to explore the functionalization of HA with active targeting ligands. Recently, we have demonstrated that HA functionalized with the tumor homing peptide tLyp-1 increases substantially the accumulation of HA NCs in a lung cancer model [28]. Following a similar approach, in this paper, we hypothesize that the functionalization of HA with ligands for receptors present on the surface of TAMs should improve their in vivo performance.

The monosaccharide mannose (Man) is one of the most well-known ligands used for the active targeting of immune cells. Man is involved in the recognition of endogenous and pathogenic molecules through the CD206 receptor, expressed by dendritic cells and macrophages [34]. Interestingly, the CD206 receptor is also a phenotypic marker overexpressed by M2-like (i.e., TAMs) versus M1-like macrophages in solid tumors [35]. In accordance, mannosylated nanocarriers have been used for the preferential targeting of TAMs [36–41]. However, very limited knowledge is still available about the combination of HA and Man in a nanostructure for TAMs targeting, which is the scope of our current work.

Here, we describe the development and evaluation of a panel of HA NCs designed to target TAMs. Different preparation techniques and compositions, including HA of different molecular weights (MWs) and Man-functionalized hyaluronic acid (HA-Man), led to a variety of prototypes which were fully characterized in terms of their physicochemical properties. Primary human macrophages were exposed to these HA NCs to test their immunotoxic activity and internalization. Finally, in order to understand their ability to reach macrophages in solid tumors, the biodistribution and tumor targeting ability of HA-Man NCs were analyzed, with and without the pre-administration of Nanoprimer™ (which decrease liver clearance of NCs) in a fully immunocompetent MN/MCA1 fibrosarcoma murine model with a high infiltration of TAMs.

## Materials and methods

### Materials

#### For the synthesis of HA-Man

All chemicals were purchased from Sigma-Aldrich and were used without further purification. Hyaluronic acid was purchased from Lifecore Biomedical (sodium hyaluronate, lot number 024168, specification number LDP-9800042; Mw 57 kDa by MALLS). 2,3,4,6-Tetra-*O*-acetyl-α-D-mannopyranosyl trichloroacetimidate and 2-[2-(2-azidoethoxy)ethoxy]ethanol were prepared following previously reported procedures [42–44]. All solvents were of HPLC grade, purchased from Scharlab and Fisher. Et_3_N was dried under 4 Å molecular sieves. CH_2_Cl_2_ was dried using a SPS800 solvent purification system from MBRAUN. H_2_O of Milli-Q grade was obtained from a Millipore water purification system. Amberlite IR-120 was sequentially washed with H_2_O, MeOH, and CH_2_Cl_2_ before use.

#### For the preparation of the NCs

Caprylic-capric triglycerides (Miglyol^®^ 812) were obtained from IOI Oleo GmbH (Hamburg, Germany). Cetrimonium bromide, DL-α-tocopherol (Calbiochem^®^), and polysorbate 80 (Tween^®^ 80) were obtained from Merck KGaA (Madrid, Spain). Benzethonium chloride was purchased from Spectrum Chemical Mfg. Corp. (New Brunswick, USA). Polyethylene glycol (15)-hydroxystearate (Kolliphor^®^ HS 15) was purchased from BASF SE (Ludwigshafen, Germany). DL-α-tocopherol-TPGS was bought to Antares Health Products Inc. (Jonesborough, USA). Lecithin-soya (Epikuron^®^ 145 V) was bought to Cargill Inc. (Minnetonka, USA). Fifty and 1500 kDa HA were received from Lifecore Biomedical Inc. (Chaska, USA). In total, 330 kDa HA was received from LEHVOSS Italia Srl. (Origgio, Italy). DiD′; DiIC_18_(5) (1,1′-dioctadecyl-3,3,3′,3′-tetramethylindodicarbocyanine, 4-chlorobenzenesulfonate salt) and DiR′; DiIC_18_(7) (1,1′-dioactadecyl-3,3,3′,3′-tetramethylindotricarbocyanine iodide) were provided by ThermoFisher Scientific Inc. (Madrid, Spain). Nanoprimer™ technology was provided by Curadigm SAS (Paris, France).

### Synthesis, isolation, and characterization of HA-Man

A solution of DMTMM (55 mg, 0.199 mmol) and 3 (7.45 mg, 0.024 mmol) in 1 mM phosphate buffer (pH 6.5, 0.4 mL) was added to a solution of HA (40 mg, 0.10 mmol) in 1 mM phosphate buffer (pH 6.5, 0.9 mL). The reaction mixture was stirred at 70 °C for 2 h and then was ultrafiltered (YM5; 4 × 30 mL sat NaHCO3, 3 × 30 mL H2O) and lyophilized to afford HA-Man (37.5 mg, DS 15.6%; coupling yield 65%; mass recovery 85%). 1H NMR (500 MHz, Dfilter 120 ms, D2O) δ: 4.90 (s, 0.16H), 4.54 (br s, 1H), 4.45 (br s, 1H), 4.05–3.25 (m, 12.81H), 2.02 (s, 3H). For more details about the synthesis of new compounds, see the supplementary materials section.

#### Column chromatography

Automated column chromatography was performed on a MPLC Teledyne ISCO CombiFlash RF 200 psi with RediSep Rf columns refilled with silica 40–63 µm (from VWR Chemicals) or neutral alumina oxide 60 mesh (from Fisher Scientific). Samples were adsorbed onto silica or neutral alumina and loaded into solid cartridges.

#### NMR spectroscopy

NMR spectra were recorded on a Varian Mercury 300 MHz or Varian Innova 500 MHz spectrometers. Chemical shifts are reported in ppm (δ units) downfield from internal tetramethylsilane (CDCl_3_), internal 3-(trimethylsilyl)propionic-2,2,3,3-*d*_4_ acid sodium salt (D_2_O), or the residual HOD peak (D_2_O). Determination of the substitution degree of HA-Man was done by relative integration between the 3H of the *N*-acetyl group of HA (2.02 ppm) and the anomeric proton of the Man pendants (4.90 ppm) in the ^1^H NMR spectrum of the polymer (D_2_O) recorded with a ^1^H-diffusion filter (stimulated Echo-LED pulse sequence with bipolar PFG gradients, relaxation delay (d1) was set to 15 s, and diffusion delay (Δ) was set to 120 ms). MestReNova 14.2 software (Mestrelab Research) was used for spectra processing.

#### Infrared spectroscopy

FT-IR spectra were recorded on a PerkinElmer spectrum two spectrophotometer equipped with an ATR accessory.

#### Mass spectrometry (MS)

Mass spectra were recorder on a Bruker Microtof spectrometer coupled to a HPLC Agilent 1100 using atmospheric-pressure chemical ionization (APCI) or electrospray ionization (ESI). Samples were injected via flow injection analysis (FIA) using a MeOH/aqueous solution of formic acid 0.1% 1:1, flow 0.2 mL/min.

#### Ultrafiltration

Ultrafiltration was performed on Millipore Amicon stirred cells with Amicon YM5 regenerated cellulose membranes under a 5 psi N_2_ pressure.

### HA NCs preparation

#### HA SE-NCs

The preparation protocol of HA SE-NCs was adapted from a previously described protocol [33, 45]. More precisely, 0.875 mL of a 2.75 mg/mL benzethonium chloride solution in polysorbate 80 was added to 1 mL of caprylic-capric triglycerides to obtain a stock solution of the oily phase. To obtain the water phase, 0.5 mL of an aqueous solution of polyethylene glycol (15)-hydroxystearate (7.5 mg/mL); 0.25 mL of an aqueous solution of HA of 50, 330, or 1500 kDa (1.5 mg/mL); and 0.575 mL of ultrapure water were mixed into a beaker. Then, 0.175 mL of the oily phase was quickly added over the water phase (1.325 mL) under magnetic stirring. Finally, the resulting HA SE-NCs were isolated using CentripureP10 size exclusion columns (EMP Biotech GmbH.; Berlin, Germany) to a final 1.5 mL volume.

#### HA SD-NCs

The preparation protocol of HA SD-NCs has been previously described [46]. More precisely, to obtain the oily phase, the following solutions in ethanol were mixed in a microtube: 0.2 mL of DL-α-tocopherol (67.5 mg/mL), 0.2 mL of DL-α-tocopherol-TPGS (20 mg/mL), and 0.1 mL of benzethonium chloride (5 mg/mL). Then, 1 mL of an aqueous solution of HA of 50 kDa (2 mg/mL) and ultrapure water (0.5 mL) were added into a beaker to obtain the water phase. The oily phase was added quickly over the water phase under magnetic stirring to conclude the emulsification phase. Lastly, the organic solvents were removed by evaporation under the fume hood, and the final volume was adjusted to 2 mL with ultrapure water.

#### HA SD-NCs/HA-Man SD-NCs loaded with fluorescent dyes for uptake and biodistribution studies

The preparation protocols of HA SD-NCs and HA-Man SD-NCs have been previously described [28]. More precisely, 0.2 mL of an 18.75 mg/mL ethanolic solution of lecithin-soya, 0.05 mL of a 15 mg/mL ethanolic solution of cetrimonium bromide caprylic-capric triglycerides (0.015 mL), and acetone (4.75 mL) were added into a test tube to obtain the oily phase. The water phase, formed by 10 mL of non-modified or mannose-modified HA of 50 kDa (0.25 mg/mL), was placed into a beaker. The oily phase was added over the water phase drop by drop under magnetic stirring. Finally, the organic solvents were removed with a rotatory evaporator and the final volume adjusted with ultrapure water up to 5 mL. When needed, HA SD-NCs and HA-Man SD-NCs were labelled with DiD or DiR, incorporating the fluorophores into the oily phase with a final concentration of 25 µg/mL.

### Physicochemical characterization of HA NCs

#### DLS

HA NCs were characterized by dynamic light scattering (DLS) using a Zetasizer Nano ZS ZEN 3600 equipment (Malvern Panalytical Ltd.; Malvern, UK). Previous to their characterization, HA NCs were diluted in ultrapure water (HA SE-NCs 1:100; HA/HA-Man SD-NCs 1:20). The measurement parameters were the same for all the samples (laser incident angle 173°, 25 °C, 6 runs, 12 s per run, 3 measurements).

### NTA

Selected HA NCs were characterized by nanoparticle tracking analysis (NTA) using a NanoSight NS300 equipment (Malvern Panalytical Ltd.; Malvern, UK). Previous to their characterization, HA NCs were diluted in ultrapure water (HA SE-NCs 1:100; HA/HA-Man SD-NCs-TG 1:20). The measurement parameters were the same for all the samples (laser wavelength 488 nm, 25 °C, 5 captures, 60 s per capture).

### STEM

Scanning transmission electron microscopy (STEM) images were taken from selected HA NCs. Firstly, HA NCs were diluted in ultrapure water (HA SE-NCs 1:10,000; HA/HA-Man SD-NCs-TG 1:2,000) and mixed with the same volume of a 2% (w/v) phosphotungstic acid solution. Then, samples were located on copper grids with carbon films and washed with 1 mL of ultrapure water. Once the grids were dried, they were observed in the microscope (FESEM; ZEISS, ULTRA Plus, Germany).

### Colloidal stability of HA NCs

#### Colloidal stability at storage conditions

The main physicochemical properties of the HA NCs were evaluated as described in the “DLS” section for 14 days during the storage of the HA NCs at 4 °C.

#### Colloidal stability in relevant biological mediums

The main physicochemical properties of the HA NCs, incubated with RPMI/10% fetal bovine serum (FBS) for 24 h, were evaluated as described in the “DLS” section.

### Endotoxin assessment

The endotoxin content of all the NCs was evaluated with PYROGENT™ Plus Gel Clot LAL and Kinetic-QCL^®^ Kinetic Chromogenic LAL (Lonza Group Ltd.; Basel, Switzerland). Endotoxin content for all samples was below the limit of 0.125 EU/mL.

### Isolation and differentiation of HMDM

Human monocyte-derived macrophages (HMDM) were isolated from healthy blood donors, as previously described [47]. HMDM were obtained by two-step gradient centrifugation using lymphocyte-H cell separation media (Merck KGaA.; Milan Italy) and Percoll™ (ThermoFisher Scientific Ins; Milan Italy). M0 macrophages were differentiated by culturing 1 × 10^6^ HMDM in RPMI/5% FBS with 25.00 ng/mL of recombinant human monocyte colony-stimulating factor-1 (rhM-CSF-1) (PeproTech Inc.; London, UK). M1 macrophages were differentiated by stimulating M0 in RPMI/5% FBS with 100 ng/ml of lipopolysaccharide (LPS) and 50 ng/mL of interferon-gamma (IFN-γ) (PeproTech Inc.; London, UK). Lastly, M2 macrophages were differentiated by stimulating M0 in RPMI/5% FBS with 20 ng/mL of interleukin-4 (PeproTech Inc.; London, UK).

### Cytotoxicity assays

HMDM were seeded in a 96-wells plate at a density of 1 × 10^5^ cells/wells. Cells were incubated with HA NCs at the indicated concentrations and times in RPMI/10% FBS. Cell viability was evaluated with AlarmarBlue™ cell viability reagent (ThermoFisher Scientific Inc.; Milan, Italy). Non-treated cells were used as 100% cell viability control.

### Expression of macrophages phenotypical markers by FACS and cytokine secretion by ELISA

M0 and M2 macrophages were seeded in a low-attachment 24-wells plate at a density of 1 × 10^6^ cells/well and incubated in RPMI/10% FBS with 100 µg/mL of HA NCs for 48 h. Treatment with IFN-γ + LPS was used as induced positive control. Cells were resuspended in fluorescence-activated cell sorting (FACS) buffer (PBS/1% bovine serum albumin (BSA)) and labelled with PerpCP™-Cy5.5 mouse anti-human major histocompatibility complex class II (MHC-II) and FITC mouse anti-human CD206 (BD Biosciences; San Jose, USA). Finally, cells were analyzed with the FACS equipment BD Canto™ (BD Biosciences; San Jose, USA). The secretion levels of the tumoral necrosis factor α (TNF-α) were measured with an ELISA kit following the instruction provided by the manufacturing company (R&D Systems Inc.; Minneapolis, USA).

### Uptake and internalization assays in macrophages

M1 and M2 macrophages were seeded in a low-attachment 24-wells plate at a density of 1 × 10^6^ cell/well. Cells were incubated with 0.50 mL of RPMI/10% FBS containing 0.5 mg/mL of DiD-labelled HA SD-NCs or DiD-labelled HA-Man SD-NCs for 1 h.

#### Flow cytometry

M1 and M2 macrophages were fixed for 20 min at 4 °C with fixative solution (PBS/4% paraformaldehyde), centrifugated, and resuspended in FACS buffer. Cells were analyzed with FACS equipment BD Canto™ (wavelengths: λ_exc_ 640 nm, λ_ems_ 670 nm).

#### Confocal microscope

M1 and M2 macrophages were seeded in a 24-wells plate with coverslips (14 nm). Cells were labelled with DAPI and fixed for 20 min at 4 °C with fixative solution (PBS/4% paraformaldehyde). Glass coverslips were recovered, mounted, and analyzed with a confocal microscope Leica TCS SP8 SMD (Leica Camera Ag.; Wetzlar, Germany) (wavelengths: λ_exc_ 640 nm, λ_ems_ 670 nm).

### Fibrosarcoma mice model

Female 6-week-old C57BL/6 mice were used as animal model. An orthotopic immunocompetent fibrosarcoma model was generated as previously described [48]. 1 × 10^5^ MN/MCA1 cells were injected into the caudal thigh muscle. Procedures involving animals were conducted following Italian (4D.L.N.116, G.U., supplement 40,18–2-1992) and European (Directive 2010/63/EU) normative. Efforts were made to minimize the number of animals used and their suffering.

### Biodistribution imaging

Mice were shaved to avoid the interferences of the fur and fed with an imaging diet. 0.10 mL of DiR-labelled HA SD-NCs and DiR-labelled HA-Man SD-NCs were administered by intravenous injection. Nanoprimer™ was injected at 360 mg/kg; 10 min before NCs administration following manufacture protocol (Curadigm SAS; Paris, France). Ketamine and xylazine at a concentration of 100 mg/kg and 10 mg/kg were used for anesthesia. Finally, mice were sacrificed with CO_2_.

#### Analysis of IVIS images

Imaging analysis was performed with IVIS equipment Lumina III System (PerkinElmer Inc; Waltham, USA). Mice were inserted in a supine position into the equipment. Tissues of interest were removed and analyzed ex vivo. IVIS equipment was used with the same configuration for all the mice and tissues (wavelengths: λ_exc_ 750 nm, λ_ems_ 780 nm; binning 4 or 8; f/stop 2).

### FACS analysis of excised tumors

Tumors were collected and prepared for FACS analysis. Cells were stained with LIVE/DEAD^®^ Fixable Aqua Dead Cell Stain (Invitrogen; 1:1000 in PBS -/-) for 30 min at room temperature (RT) and then stained with the mix of antibodies (CD45-PerCP—Clone 30-F11 (BD Biosciences); Cd11b-APC eFluor780—Clone M1/70 (eBiosciences); F4/80-PE—Clone Cl:A3-1 (BioRad)), in FACS buffer for 30 min at 4 °C. Cells were washed with FACS buffer and fixed with FACS fix Buffer (1% PFA PBS) for 20 min at 4 °C. Cells were analyzed on a FACS Canto™ II analyzer and data generated by FloJow software (BD Biosciences).

### Statistical analysis

Statistical analysis was performed with GraphPad Prism software. Statistical comparisons were done using two-way ANOVA with Turkey’s multiple comparison test and one-way with Turkey’s multiple comparison test. Data were expressed as the mean ± standard deviation. *P* values 0.05 or less were considered statistically significant. *(*p* < 0.05); **(*p* < 0.01); ***(*p* < 0.001); ****(*p* < 0.0001).

## Results and discussion

### Design, preparation, and characterization of HA NCs

**Fig. 1:**
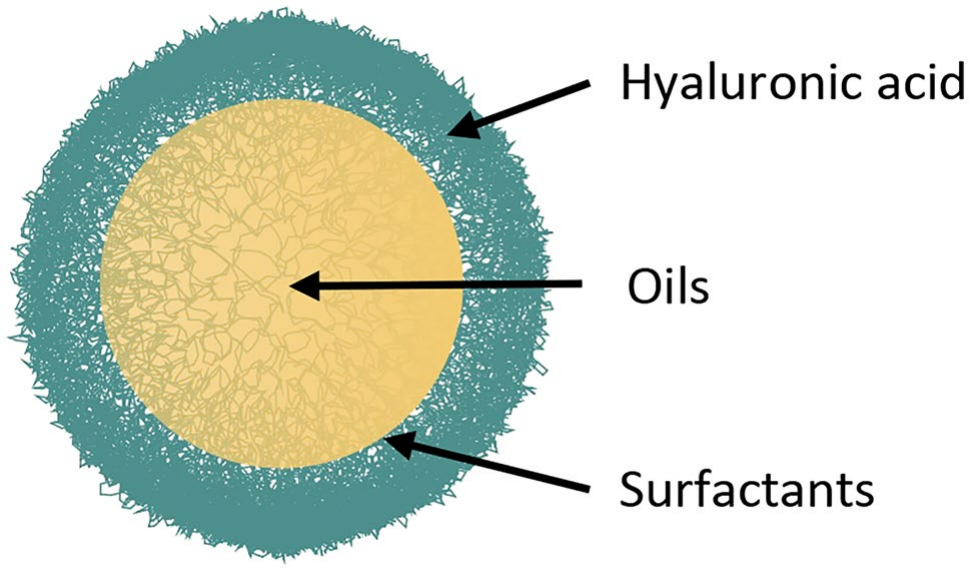
Illustration of the hyaluronic acid nanocapsules (HA NCs)

As a first step towards the development of HA NCs functionalized with mannose for the targeting of TAMs, we have prepared a series of NCs with HA of different molecular weights (MWs), using different preparation techniques and compositions. In order to do this, we have built on the experience of our research group in the design and synthesis of polymeric nanocapsules (NCs) by solvent displacement (SD) or self-emulsification (SE) techniques [49–52]. As a continuation of our previous work, here we study the relevance of the formulation parameters and composition of HA NCs, including their functionalization with mannose, for their interaction with macrophages in the tumor microenvironment. We developed a panel of HA NCs by the SE technique using HA of different MWs (50, 330, 1500 kDa) (Fig. 1). The polymeric coating and an oily core of caprylic-capric triglycerides of these prototypes (HA-50/HA-330/HA-1500 SE-NCs) were similar to the ones that have already shown good intracellular mAb delivery [33, 45]. The counterpart nanoemulsions, without the polymeric coating (SE NEs), were included as control. Moreover, based on our previous work using docetaxel-loaded nanosystems [28], NCs prepared by the SD technique with a 50 kDa HA polymeric coating and an oily core of DL-α-tocopherol (HA SD-NCs) [46] were also included in the panel (Table S1).

**Fig. 2:**
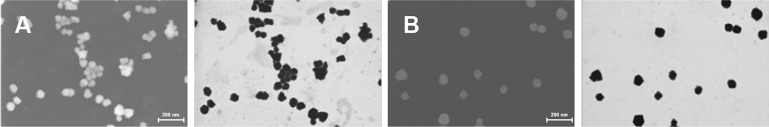
STEM images of **A** SE-NEs; **B** HA-50 SE-NCs. EHT = 20.00 kV; WD = 2.4 mm Mag = 200.00 K X. EHT, electron high tension; Mag, magnification; STEM, scanning transmission electron microscopy; WD, work distance

All HA NCs showed similar physicochemical properties, with a particle size between 120 and 150 nm (PDI 0.2–0.3) and Z-potential ranging from slightly negatively charged, for HA SE-NCs, to highly negative, for HA SD-NCs (Table 1) [53]. A deeper analysis of selected prototypes from the SE NCs group was performed, because due to their more recent development by our group, they lack the SD NCs exhaustive characterization. Thus, HA-50 SE-NCs and their corresponding NE were characterized with orthogonal techniques, such as NTA and STEM, following the EU-NCL recommendations [54]. These techniques added relevant information about particle size, size distribution, and particle shape [55]. The HA-50 SE-NCs and the corresponding nanoemulsions (SE-NEs) showed similar particle size and distribution (Table 2). Moreover, STEM images showed particles with a spherical shape around 100 nm (Fig. 2). Finally, the whole panel of HA NCs were characterized regarding their stability in storage conditions and relevant biological medium. Our results show that the HA NCs were stable at 4 °C for at least 15 days and in RPMI/10% FBS at 37 °C for, at least, 24 h (Fig. S1).

**Table 1:** DLS characterization of HA NCs (*n* = 3)

Nanocarrier	Particle size (nm)	PDI	Zeta potential
SE-NEs	139 ± 13	0.25	+ 3 ± 1 mV
HA-50 SE-NCs	130 ± 4	0.25	− 13 ± 3 mV
HA-330 SE-NCs	133 ± 3	0.25	− 18 ± 2 mV
HA-1500 SE-NCs	123 ± 5	0.25	− 19 ± 3 mV
HA SD-NCs	151 ± 8	0.15	− 36 ± 3 mV

**Table 2:** NTA characterization of selected HA NCs (*n* = 3)

Nanocarrier	Mean (nm)	σ (nm)	D10 (nm)	D50 (nm)	D90 (nm)	Particles per mL
SE-NEs	115 ± 6	44	74	104	168	9·10^12^ ± 2·10^12^
HA-50 SE-NCs	108 ± 5	37	72	99	144	6·10^12^ ± 2·10^12^

*D10/50/90* distribution of the 10%/50%/90% of the particle population, *DLS* dynamic light scattering, *HA SD-NCs* nanocapsules prepared by solvent displacement with a 50-kDa HA polymeric coating and an oily core of DL-α-tocopherol, *HA SE-NCs* nanocapsules prepared by self-emulsifying with a HA polymeric coating, *NTA* nanoparticle tracking analysis, *PDI* polydispersity index, *SE-NEs* nanoemulsion prepared by self-emulsifying without polymeric shell, *σ* standard deviation

### Toxicological evaluation of HA NCs

**Fig. 3:**
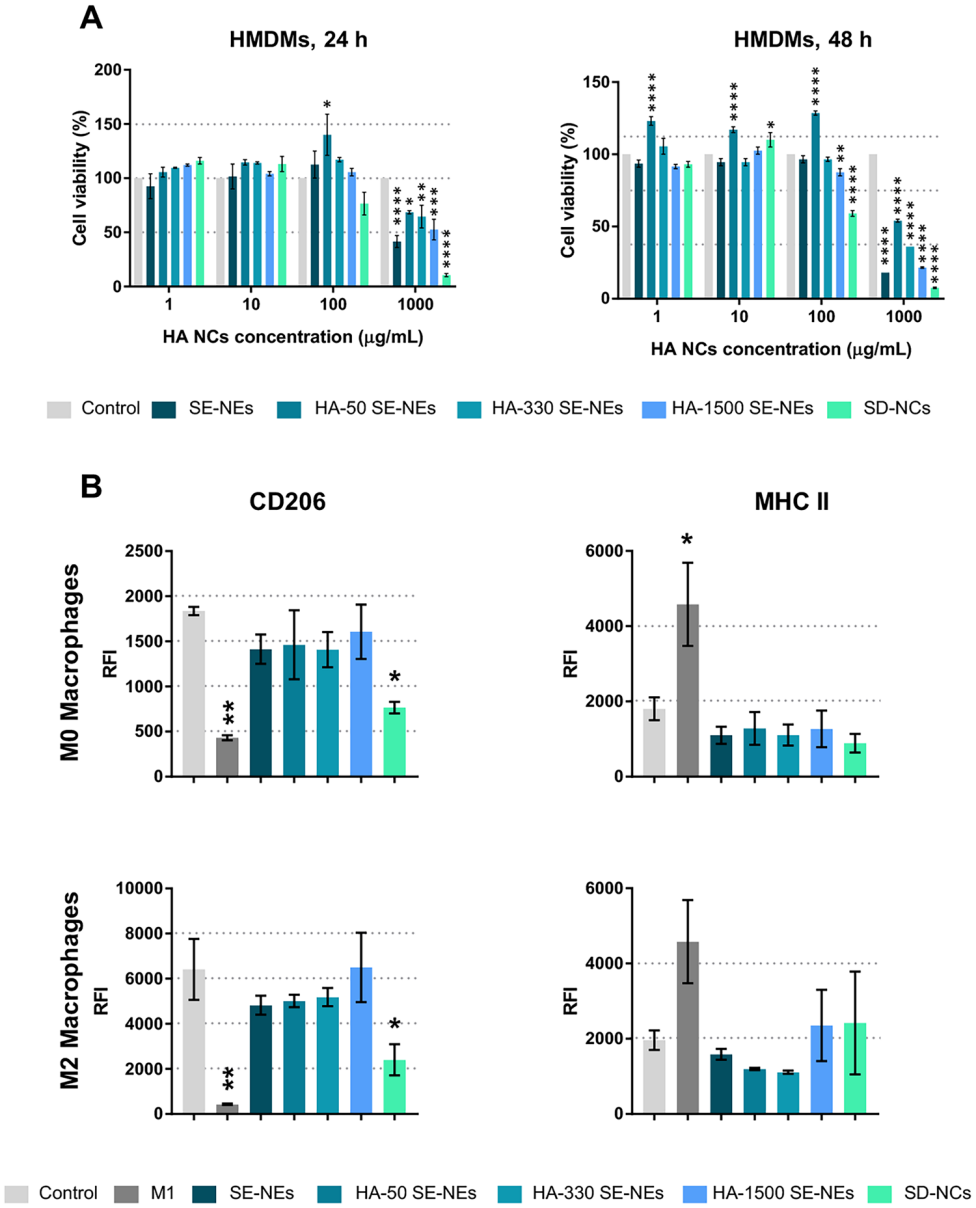
**A** HA NCs toxicity profile towards HMDM at 24 and 48 h; AlamarBlue™ cell viability assay. **B** Quantification of CD206 (M2 phenotypic marker) and MHC-II (M1 phenotypic marker) on the surface of M0 and M2 macrophages after 48-h incubation with HA NC, measured by FACS. M1 macrophages (treated with IFN-γ + LPS) were used as positive control. Statistical comparison was performed using one-way and two-way ANOVA with Tukey’s multiple comparison test. *(*p* < 0.05); **(*p* < 0.01); ***(*p* < 0.001); ****(*p* < 0.0001) respect to control group. RFI, relative fluorescence intensity

To evaluate the toxicity of the nanostructures in vitro, human monocyte–derived macrophages (HMDMs) were exposed to different concentrations of HA NCs for 24 and 48 h. All nanosystems showed good tolerability up to a concentration of 100 µg/mL. Only the NCs prepared by the solvent displacement technique (HA SD-NCs) at a concentration of 100 µg/mL showed a minor toxicity, presenting a half-maximal inhibitory concentration (IC-50) of 209.7 µg/mL at 24 h and 116.3 µg/mL at 48 h (Fig. 3A) (Table S2). The higher toxicity of these HA SD-NCs is likely related to the content of cationic surfactant in their structure [56]. All HA SE-NCs, prepared with HA of different MWs, showed similar toxicity at 24 h, with IC-50 around 1000 µg/mL (Fig. 3A) (Table S2). Overall, these results demonstrate the good biocompatibility of all the developed HA NCs and allow for the selection of non-toxic doses for further in vitro experiments as described below.

### Effect of HA NCs on the phenotype and polarization of macrophages

With regard to the immunomodulatory properties of HA, others have suggested a pro-inflammatory activity for low molecular weight HA (< 150 kDa) versus anti-inflammatory properties for high molecular weight HA (> 1000 kDa) [57]. Thus, we decided to test the immunomodulatory properties of our HA NCs series (using HA of 50, 330, and 1500 kDa). For this, primary human macrophages, M0 (non-polarized) and M2 (IL-4 treated) HMDMs, were exposed for 48 h to the HA NCs. Incubation of HMDMs with IFN-γ + LPS was used as positive control of M1-like polarization (antitumor macrophages). The CD206 (mannose receptor) was quantified as M2-like marker, while, as indication of M1-like polarization, the amount of MHC-II receptor on the surface of macrophages was measured by FACS. The results in Fig. 3B indicate that none of the HA SE-NCs induced significant variations in the expression levels of the CD206 and MHC-II receptors in M0- or M2-treated macrophages. However, M0 and M2 macrophages exposed to HA SD-NCs showed a significant reduction in the amount of CD206 receptor, which could be related to the toxicity of this formulation at 48 h (Fig. 3A, B). Overall, no significant variations were observed in the MHC-II receptor for any of the HA NCs in M0- or M2-exposed macrophages, indicating no ability to induce M1-like polarization (as observed for the positive controls). To validate these results, the secretion of TNF-α by M0 and M2 macrophages incubated for 48 h with 100 µg/mL of the different HA NCs series was evaluated by ELISA. None of the formulations tested was able to induce the secretion of this pro-inflammatory cytokine (Fig. S2). These results are in agreement with previous research by Mizrahi et al. [58] that showed that multilamellar vesicles coated with polymeric HA of different MWs did not affect the differentiation of RAW 264.7 macrophages. As a whole, our experiments using human primary macrophages indicate that neither the MW of HA nor the different surfactants used in the SE or the SD techniques (see detailed composition in Table S1) significantly affected the polarization of macrophages.

### Synthesis of mannosylated hyaluronic acid (HA-Man) and development of HA-Man NCs

To improve the ability of HA NCs to target macrophages in the TME, we implemented the functionalization of the NCs with mannose, as a targeting ligand. It is well-known that the mannose receptor (CD206) is overexpressed in TAMs (with an M2 phenotype) versus in M1 and M0 (non-polarized) macrophages [2]. Thus, we synthetized a new HA polymer functionalized with mannose (Man) residues, as ligands to be exposed on the surface of the NCs for the active targeting of the CD206 receptors on the surface of TAMs.

**Fig. 4:**
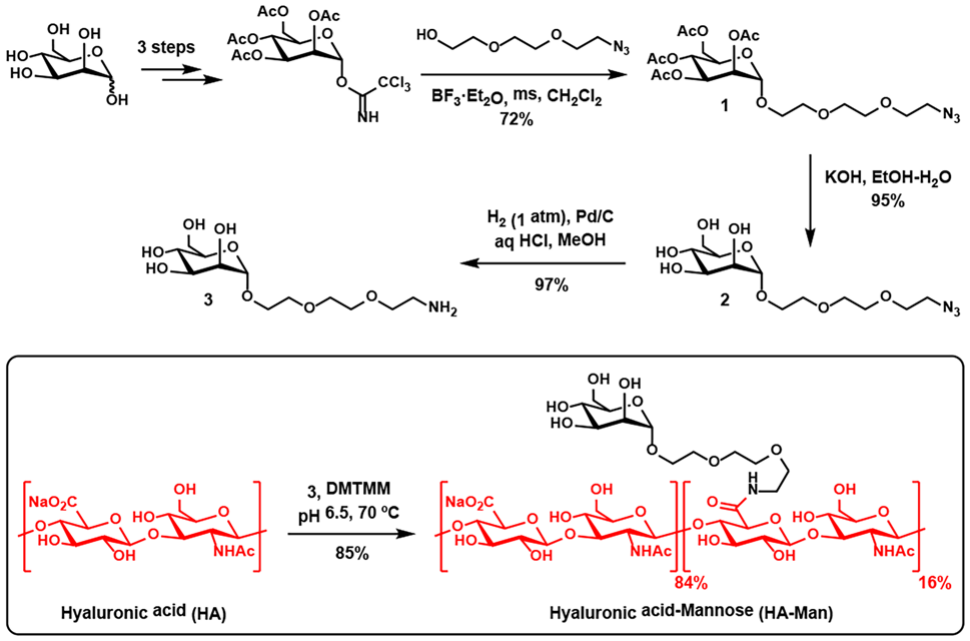
Synthesis of mannosylated hyaluronic acid (HA-Man)

The mannosylated HA (HA-Man) was produced by amide coupling between the carboxylic acid at the D-glucuronic acid monomers of HA and an aminated mannose derivative (3), provided with a flexible and hydrophilic linker to ensure an effective receptor-mediated recognition by the corresponding cells (Fig. 4). The amine 3 was prepared in three steps from an acetylated mannose trichloroacetimidate precursor [43, 59]. First, a BF_3_-catalyzed glycosylation with 2-[2-(2-azidoethoxy)ethoxy]ethanol afforded the α-mannose derivative **1** in 72% yield. The α configuration at the anomeric position of **1** was confirmed by ^1^H NMR according to the observed *J* = 1.7 Hz coupling constant (see supplementary information). Deprotection of the acetyl groups (KOH, MeOH, 95%), followed with azide reduction by hydrogenation (1 atm, Pd/C, 97%) afforded 3 in excellent yield. Amide coupling between HA (50 kDa) and amine **3** was performed under slightly acidic aqueous conditions in the presence of 4-(4,6-dimethoxy-l,3,5-triazin-2-yl)-4-methylmorpholinium chloride (DMTMM) [42], a water-stable triazine-based condensing reagent. Interestingly, the yield using DMTMM was superior to the one obtained when using EDC/NHS [60]. The resulting HA-Man was obtained with an 85% mass recovery after purification by ultrafiltration. A degree of substitution (DS) of 16% was determined by relative integration in the ^1^H NMR spectrum of the polymer (D_2_O) between the *N*-acetyl protons of the HA chain (2.02 ppm) and the anomeric proton of the mannose pendants (4.90 ppm) (see supplementary information). Considering that two monosaccharides comprise the repeating unit of HA, this results in a degree of substitution of 8% for our polymer on a monosaccharide basis.

**Fig. 5:**
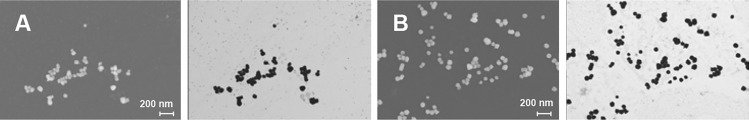
**A** HA SD-NCs images; **B** HA-Man SD-NCs STEM images. EHT = 20.00 kV; WD = 2.5 mm Mag = 100.00 K X. STEM, scanning transmission electron microscopy

Using the HA-Man polymer, we prepared NCs by the SD technique (HA-Man SD-NCs), and their physicochemical and biological properties were compared with those of the corresponding non-modified NCs (HA SD-NCs). It is worth mentioning that our research group has previously demonstrated the versatility of similar HA SD-NCs to be functionalized with peptide targeting ligands, such as t-LyP-1. The t-LyP-1-HA-NCs showed a significant improvement in their biodistribution profile, drug accumulation in the tumor (when loaded with docetaxel), and a better antitumoral efficacy in an A549 non-small lung cancer animal model [28]. In the current work, HA SD-NCs and HA-Man SD-NCs showed similar physicochemical properties, despite the Man functionalization (Table 3). HA SD-NCs and HA-Man SD-NCs were also characterized with NTA and STEM. Minor modifications in the preparation of HA SD-NCs for in vivo experiments were performed at this step, as described in the methodology “HA SD-NCs/HA-Man SD-NCs loaded with fluorescent dyes for uptake and biodistribution studies” section, and a comprehensive characterization for both formulations was completed (our results found no significant differences between them). HA-Man SD-NCs presented a 20 nm larger mean diameter and a more heterogeneous population compared to HA SD-NCs (Table 4). The mean particle size determined by NTA was smaller than the one observed with DLS for both prototypes. In both cases, STEM images showed spherical particles with a size of about 100 nm (Fig. 5).

**Table 3:** DLS characterization of HA SD-NCs and HA-Man SD-NCs

Nanocarrier	Particle size (nm)	PDI	Zeta potential (mV)
HA SD-NCs	160 ± 4	0.10	− 50 ± 7
HA-Man SD-NCs	158 ± 12	0.15	− 39 ± 7

**Table 4:** NTA characterization of HA SD-NCs and HA-Man SD-NCs

Nanocarrier	Mean (nm)	SD (nm)	D10 (nm)	D50 (nm)	D90 (nm)	Particles per mL
HA SD-NCs	97 ± 8	27	70	94	132	4·10^12^ ± 2·10^12^
HA-Man SD-NCs	117 ± 7	39	80.7	110	164	4·10^12^ ± 2·10^12^

*D10/50/90* distribution of the 10%/50%/90% of the particle population, *DLS* dynamic light scattering, *HA SD-NCs / HA-Man SD-NCs* nanocapsules prepared by solvent displacement with a mannose-modified 50-kDa hyaluronic acid polymeric coating and an oily core of caprylic-capric triglycerides, *NTA* nanoparticle tracking analysis, *PDI* polydispersity index, *SD* standard deviation

### Internalization of mannosylated and non-mannosylated HA NCs by macrophages

**Fig. 6:**
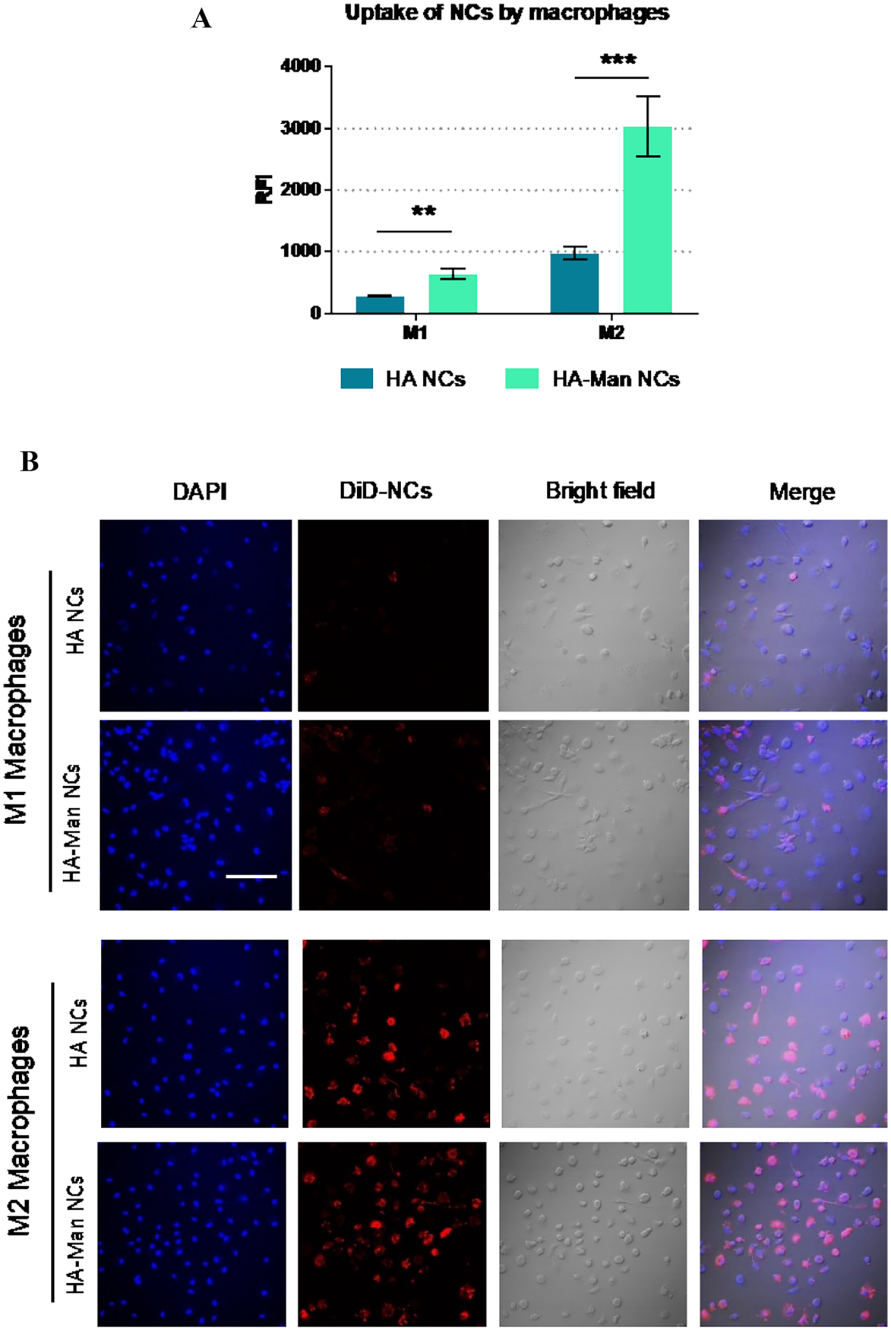
**A** Internalization assays in M2 versus M1 macrophages after their exposure to HA NCs and HA-Man NCs for 1 h. **B** Confocal microscopy images of M1 and M2 macrophages exposed to HA NCs and HA-Man NCs for 1 h; DAPI (blue), DiD-loaded NCs (red). RFI, relative fluorescence intensity. Statistical comparison was performed using two-way ANOVA with Tukey’s multiple comparisons. **(*p* < 0.01)

To evaluate their uptake by macrophages, both HA NCs and HA-Man NCs described in the previous section were now labelled with DiD, and internalization assays were evaluated by FACS and confocal microscopy. First, primary human M1 (antitumoral) and M2 (protumoral) macrophages were cultured in vitro, and the presence of the CD206 receptor on the surface of the cells for each polarization status was measured. As expected, the highest value was observed for the M2 macrophages, mimicking TAMs [35] (Fig. S3). After 1 h of exposure, HA-Man NCs were found to be internalized by macrophages of both phenotypes in a greater extent than non-functionalized HA NCs (Fig. 6A). Of note, the increased uptake was particularly remarkable for M2 macrophages (3.1-fold) (Fig. 6A). These results were validated by confocal microscopy, confirming the internalization of the NCs and showing the same pattern for the uptake of HA-Man NCs versus HA NCs by M1 and M2 macrophages (Fig. 6B).

These internalization assays using primary human macrophages showed that the functionalization of HA with mannose greatly enhanced the ability of the HA NCs to target M2 macrophages (threefold higher for M2 versus M1). Similar results have been reported for other types of nanocarriers functionalized with mannose, although the improvement of the uptake varied depending on the nanocarrier composition. For example, our results are strikingly better than others previously reported for mesoporous nanocarriers functionalized with HA-Man (Gao et al. [61]), which provided only a slight increase in the uptake by M2 macrophages (around 10% improvement). A similar improvement in uptake was observed in other studies using cell lines. Gennari et al., using XS106 dendritic cells, observed a higher uptake for chitosan/hyaluronic acid nanoparticles, only when the mannose was presented on their surface with the proper mode of ligand presentation [62]. In another work, Mahor et al. developed mannosylated polyethyleneimine-hyaluronan nanohybrids for gene delivery and found a high transfection rate through the endocytic pathway mediated by the mannose receptor in RAW264.7 and THP-1 cell lines [63]. Our results, using primary human macrophages, confirm the efficacy of HA-Man NCs to target preferentially the M2 macrophages in vitro and encourage further experiments to test their ability to target TAMs in vivo.

### Biodistribution and tumor targeting ability of mannosylated and non-mannosylated HA NCs

Numerous nanotechnological approaches have been investigated to improve the delivery of drugs to the tumor site, so as to improve their antitumoral activity and reduce their side effects. Most of these experiments have been performed with immunodeficient murine models that are commonly used in preclinical biomedical research and present significant differences when compared with a fully functional immune system in terms of nanoparticle clearance and biodistribution [6, 64]. Another issue related to the intravenous administration of nanocarriers is their high hepatic clearance: The liver, acting as a “physiological filter,” hampers their ability to reach the tumor [65, 66]. Taking these considerations into account, we have chosen the MN/MCA1 orthotopic fibrosarcoma murine model, fully immunocompetent (C57BL/6 mice), presenting a high infiltration of TAMs and high vascularization [48] as the optimal model to evaluate in vivo the TAM-targeting capacity of the HA NCs, with and without mannose, upon intravenous administration. To address the challenge presented by the liver accumulation, we have also tested the biodistribution of NCs after the pre-administration of the liposomal liver occupying agent Nanoprimer™ [67]. Nanoprimer™ blocks temporarily the uptake by the hepatic cells, mainly Kupffer cells and liver sinusoidal endothelial cells, and thus provides a therapeutic window for the administration of the polymeric NCs and improves their accumulation in the tumor.

**Fig. 7:**
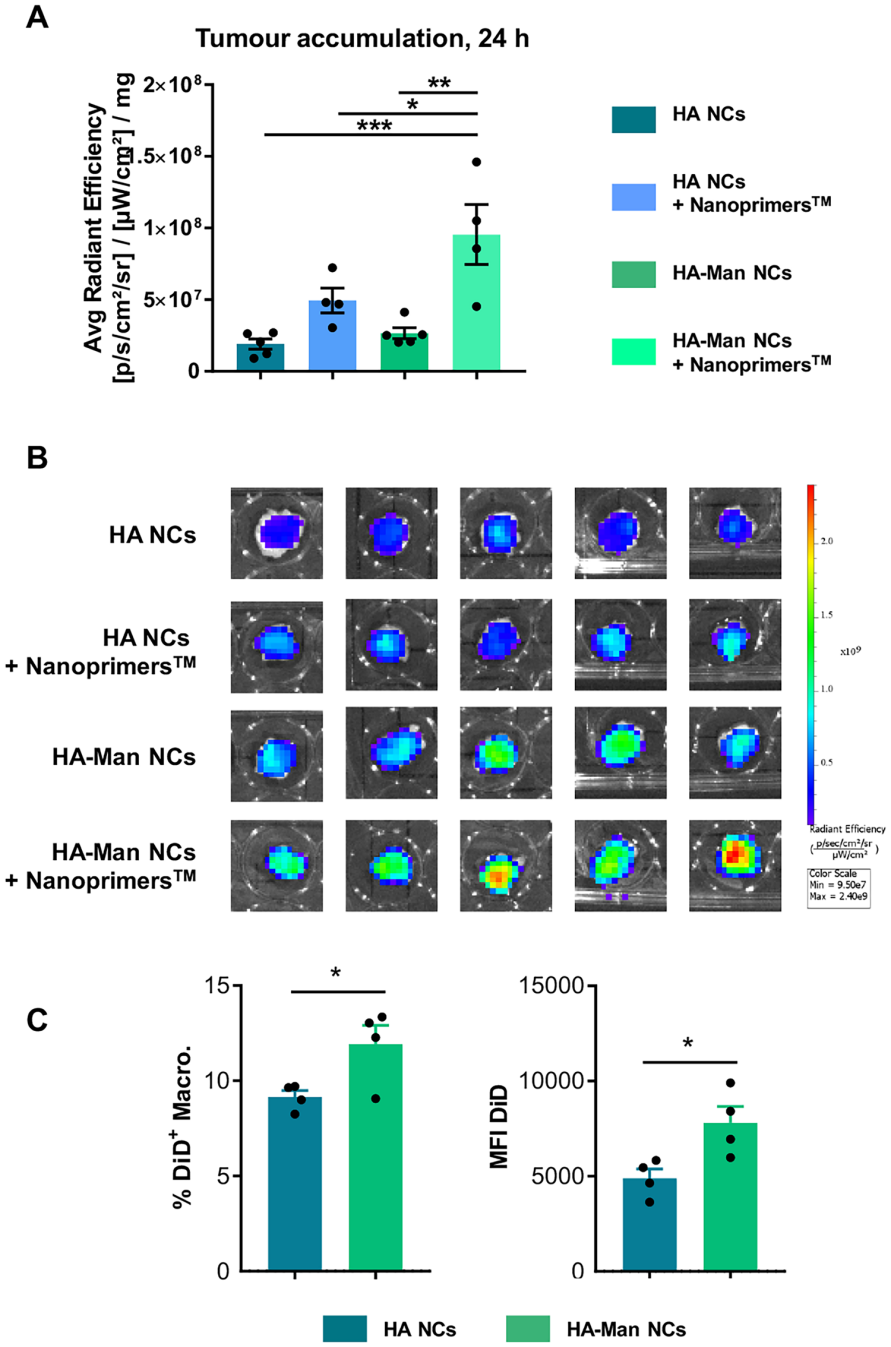
Tumor accumulation of HA and HA-Man NCs, 24 h after their intravenous administration in a MN/MCA1 fibrosarcoma mouse model. **A** Ex vivo fluorescence signal of NCs loaded with DiR in the excised tumor corrected by milligrams of tissue. Liposomal liver buffering agent (Nanoprimer™) was administered 10 min before the intravenous (i.v.) injection of the NCs. **B** Ex vivo images of the excised tumors at 24 h. **C** HA and HA-Man NCs accumulation in macrophages (CD45^+^; CD11b^+^; F4/80^+^) sorted by FACS from excised tumors: percentage of positive cells (DID.^+^ cells) and mean fluorescence intensity. Statistical comparison was performed using one-way ANOVA with Tukey’s multiple comparison test. *(*p* < 0.05), **(*p* < 0.01), ***(*p* < 0.001)

In agreement with this experimental rationale, it was found that, 24 h after intravenous injection, the HA-Man NCs had a significantly higher access to the tumor than the non-functionalized HA NCs (Fig. 7A, B). Additionally, it was observed that the administration of the Nanoprimer™ (injected 10 min before the NCs) was able to increase the accumulation of both HA-Man and HA NCs in the tumor (1.41- and 4.48-fold increase, respectively) (Fig. 7A, B). This strategy meant to reduce liver uptake following intravenous administration has been previously validated for other types of nanocarriers (i.e., loaded with siRNA) designed to target cancer cells [68]. As expected, this enhanced accumulation was attributed to the 1.30-fold increase enhanced uptake of the functionalized NCs by the TAMs (Fig. 7C). By FACS analysis of ex vivo excised tumors, it was observed a higher accumulation of HA-Mannose NCs, versus the HA NCs, in the macrophages present in the fibrosarcoma tumors. Both higher number of macrophages (CD45^+^; CD11b^+^; F4/80^+^) loaded with the fluorescent dye (DiD) and higher mean fluorescence intensity were observed for the functionalized HA-Mannose NCs (Fig. 7C).

**Fig. 8:**
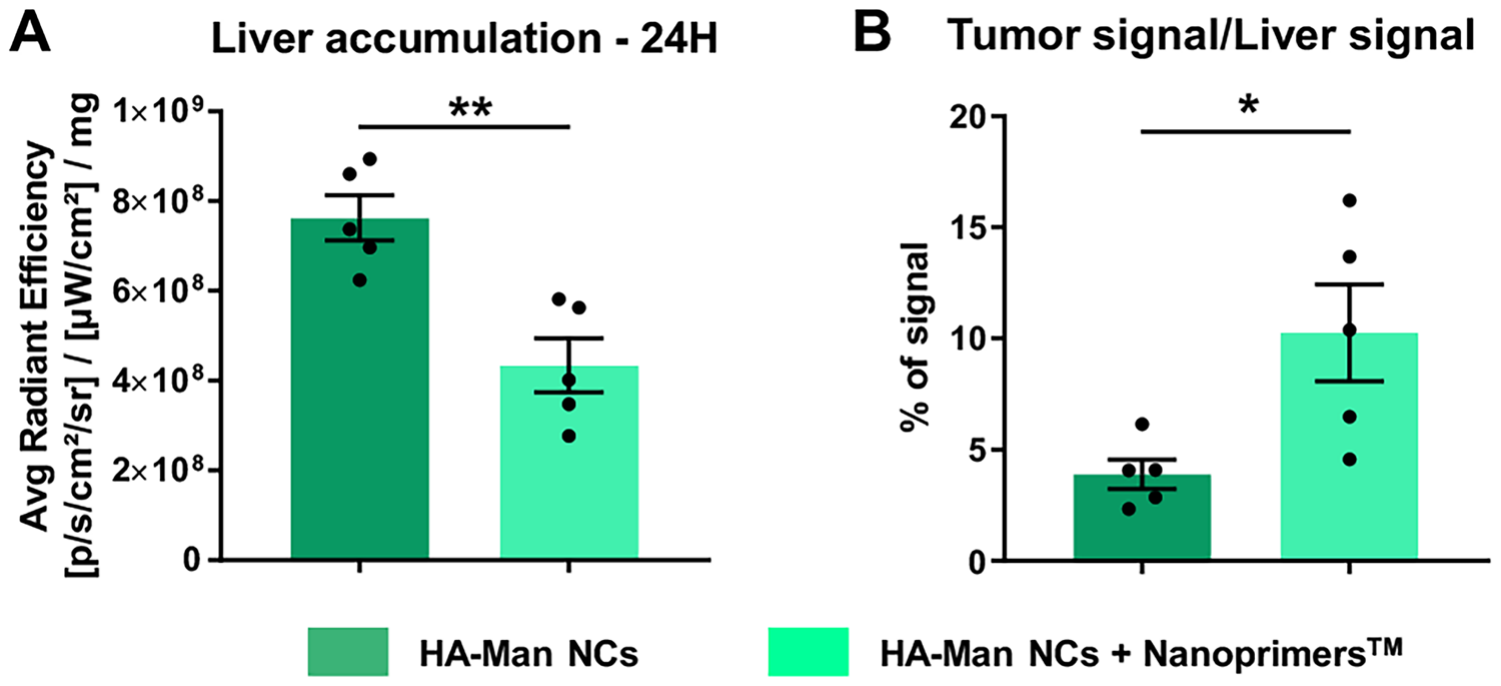
Accumulation of HA-Man NCs in the liver or tumor, with or without Nanoprimer.™ pre-administration, 24 h after their intravenous injection. **A** Ex vivo fluorescence signal (average radiant efficiency) from the whole tumor normalized to the signal from the whole liver in the MN/MCA fibrosarcoma murine model is presented as percentage. **B** Ex vivo fluorescence signal (average radiant efficiency) in the liver, related to the accumulation of HA-Man NCs corrected by milligrams of tissue in healthy mice (statistical comparison was performed using t-test. *(*p* < 0.05), **(*p* < 0.01)

For a more detailed analysis of the biodistribution profile, HA-Man NCs were administered intravenously, with and without Nanoprimer™, in tumor-free mice. Lower accumulation of HA-Mannose NCs was confirmed in the liver following pre-administration of the Nanoprimer™ by ex vivo analysis (average radiant efficiency of the whole tissue) after the experiment was completed (24 h) (Fig. 8A). During the first hour after the administration of the HA-Man NCs, a significantly lower accumulation was observed in the liver of mice pre-treated with Nanoprimer™ (1.76-fold), and this effect persisted over time, resulting in lower liver accumulation after 24 h also in these “healthy” animals (Fig. S4A and B). These dedicated experiments confirmed our results on the fibrosarcoma tumor model, where a significant improvement in the ratio of tumor/liver accumulation of HA-Man NCs was observed with Nanoprimer™ pre-treatment from 3.90 to 10.26% (Fig. 8B).

Other mannose-functionalized nanosystems of different compositions have been previously tested, mostly using partially immunodeficient murine tumor models, for the targeting of TAMs [41, 61]. Gao et al. demonstrated higher tumor accumulation for mannose-HA-mesoporous calcium silicate nanocomposites (MCNs) versus the same MCNs not coated by HA-Man, in xenograft models, consisting on 4T1 breast cancer cells injected in balb/c mice [61]. However, in this case, undesirable higher liver accumulation was also observed for the MCNs when coated with HA-Man. Using similar tumor models, Song et al. tested the biodistribution of pH-sensitive poly(allylamine hydrochloride)-based nanoparticles coated with PEG and mannose double-modified trimethyl chitosan [41]. Their results demonstrated a satisfactory tumor accumulation, which was correlated with low liver uptake and prolonged blood circulation using these nanostructures. Now, as a novelty, in the present study, we have used fully immunocompetent mice (C57BL/6) with orthotopic fibrosarcoma tumors and showed the efficacy of the functionalization of HA with mannose as a strategy to improve the ability of polymeric nanocapsules to reach TAMs in solid tumors.

## Conclusions

We have developed a simple method to produce hyaluronic acid (HA) nanocapsules, using different preparation techniques, starting with HA of different MWs and functionalizing their surface with mannose for the preferential targeting of macrophages in solid tumors. Our results demonstrate the biocompatibility and safe profile of these versatile NCs, which allows the easy combination of different components and is not affected by the MW of the polymer or the preparation techniques. In contrast to previous reports on the capacity of HA to affect the polarization of macrophages [57], the NCs evaluated in this study did not show an immunomodulatory activity in primary human macrophages, encouraging their use for biomedical applications. Furthermore, the HA NCs functionalized with mannose were able to reach TAMs in solid tumors. A clear improvement in the biodistribution profile of the HA-Man NCs after intravenous administration was observed, with a higher blood circulation time, lower hepatic uptake, and significant increase in tumor accumulation. The combination of the HA-Man NCs with Nanoprimer™ increased, even more, the tumor targeting ability of the HA-Man NCs in a fibrosarcoma tumor model. Therefore, these results provide a series of versatile HA nanosystems for the targeting of macrophages in solid tumors, with potential to be loaded with appropriate cargo (i.e., pharmacological molecules), that could be used for prognosis or therapeutic purposes.

### Supplementary Information

Below is the link to the electronic supplementary material.

Supplementary file1 (DOCX 1034 kb)

## Data Availability

All data are available upon reasonable request.

## Abbreviations

*BSA*: Bovine serum albumin
*DLS*: Dynamic light scattering
*FACS*: Fluorescence-activated cell sorting
*FBS*: Fetal bovine serum
*HA*: Hyaluronic acid
*HA-50*: Hyaluronic acid of 50 kDa
*HA-330*: Hyaluronic acid of 330 kDa
*HA-1500*: Hyaluronic acid of 1500 kDa
*HA NCs*: Nanocapsules with a hyaluronic acid polymeric coating
*HA SD-NCs*: Nanocapsules prepared by a solvent-displacement technique
*HA SE-NCs*: Nanocapsules prepared by a self-emulsifying technique
*HA-Man*: Mannose-functionalized hyaluronic acid
*HDMD*: Human monocyte-derived macrophages
*IC-50*: Half-maximal inhibitory concentration
*IL*: Interleukin
*IFN-γ*: Interferon gamma
*LPS*: Lipopolysaccharide
*Man*: Mannose
*MHC-II*: Major histocompatibility complex class II
*NCs*: Nanocapsules
*NTA*: Nanoparticle tracking analysis
*PDI*: Polydispersity index
*rhM-CSF*: Recombinant human monocyte colony-stimulating factor
*SE-NEs*: Nanoemulsion prepared by self-emulsifying technique
*STEM*: Scanning transmission electron microscopy
*TAMs*: Tumor-associated macrophages
*TNF-α*: Tumor necrosis factor α
