# Supplementary material for: Mannose-modified hyaluronic acid nanocapsules for the targeting of tumor-associated macrophages
Source: Drug Deliv Transl Res. 2022 Dec 6;13(7):1896–911. doi: 10.1007/s13346-022-01265-9 (PMC10238357; doi:10.1007/s13346-022-01265-9)
Supplement: Supplementary file 1 — Supplementary file1 (DOCX 1034 kb) [file 13346_2022_1265_MOESM1_ESM.docx]

**A. Supplementary**

| **Table S1**. Theoretical quantity of the different compounds (mg) per 1 mL of HA NCs. | | | |
| --- | --- | --- | --- |
| Component | HA SE-NCs | HA SD-NCs | HA SD-NCs  HA-Man SD-NCs  (uptake and biodistribution studies) |
| Caprylic-capric triglyceride | 59.00 | / | 2.95 |
| DL-α-tocopherol | / | 6.75 | / |
| DL-α-tocopherol-TPGS | / | 2.00 | / |
| Benzethonium chloride | 0.15 | 0.25 | / |
| Cetrimonium bromide | / | / | 0.15 |
| Lecithin-soya | / | / | 0.75 |
| Polysorbate 80 | 58.0 | / | / |
| Polyethylene glycol | 2.50 | / | / |
| Hyaluronic acid | 0.25 | 1.00 | 0.50 |
| Total | 119.90 | 10.00 | 4.35 |
| *HA SE-NCs*. Nanocapsules prepared by self-emulsifying with a hyaluronic acid polymeric coating. *HA SD-NCs (in vitro studies):* Nanocapsules prepared by solvent-displacement with a 50 kDa hyaluronic acid polymeric coating and an oily core of DL-α-tocopherol; *HA SD-NCs/ HA-Man SD-NCs (uptake and biodistribution studies)*: Nanocapsules prepared by solvent-displacement with a mannose-modified 50 kDa hyaluronic acid polymeric coating and an oily core of caprylic-capric triglycerides. 25 µg/mL of DiD for uptake *in vitro* experiments or 25 µg/mL of DiR for *in vivo* biodistribution experiments. | | | |


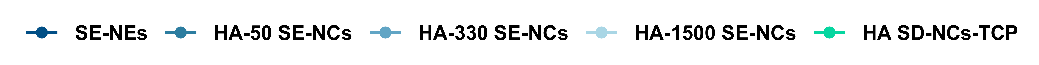

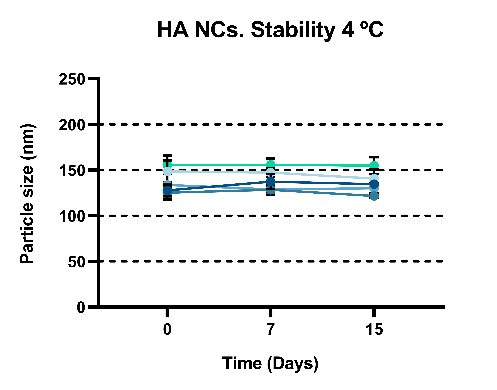

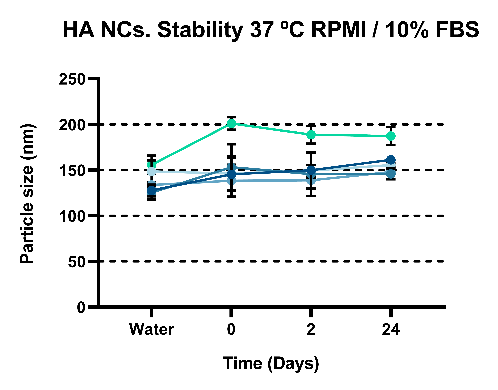


**Figure S1.** Stability of HA NCs during their storage at 4 ºC and incubation at 37 ºC in RPMI/10% FBS.

*HA SD-NCs:* Nanocapsules prepared by solvent-displacement with a 50 kDa hyaluronic acid polymeric coating and an oily core of DL-α-tocopherol; *HA SE-NCs*. Nanocapsules prepared by self-emulsifying with a hyaluronic acid polymeric coating; *SE-NEs*: Nanoemulsion prepared by self-emulsifying without polymeric shell.

| **Table S2**. HMDM IC50 of the HA NCs series. | | |
| --- | --- | --- |
| Nanocarrier | IC50 24 hours (µg/mL) | IC50 48 hours (µg/mL) |
| SE-NEs | >1000 | 392.2 |
| HA-50 SE-NCs | >1000 | >1000 |
| HA-330 SE-NCs | >1000 | >1000 |
| HA-1500 SE-NCs | >1000 | 440.9 |
| HA SD-NCs | 209.7 | 116.3 |
| *HA SD-NCs:* Nanocapsules prepared by solvent-displacement with a 50 kDa hyaluronic acid polymeric coating and an oily core of DL-α-tocopherol; *HA SE-NCs*. Nanocapsules prepared by self-emulsifying with a hyaluronic acid polymeric coating; *HMDM*: Human monocyte-derived macrophages; *IC50*: Half-maximal inhibitory concentration; SE*-NEs*: Nanoemulsion prepared by self-emulsifying without polymeric shell. | | |


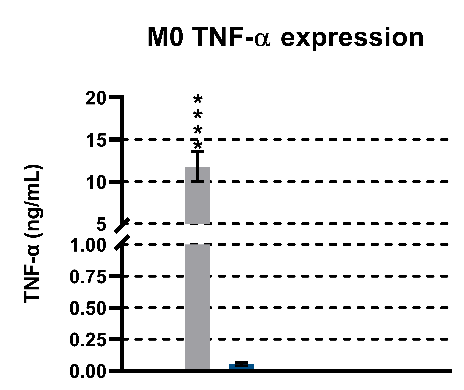

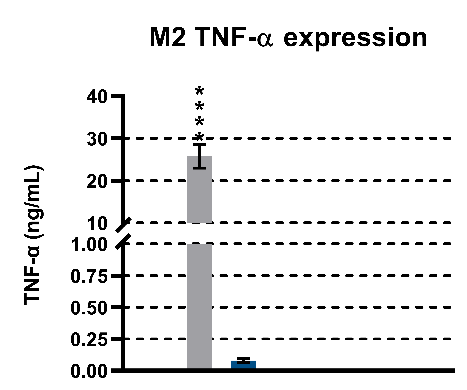

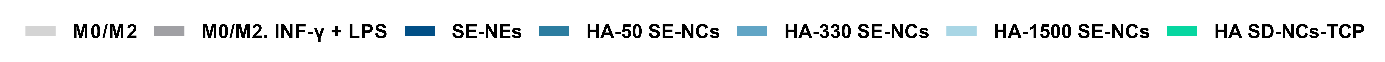


**Figure S2.** TNF-α secretion after the exposure of M0 and M2 macrophages to HA NCs for 48 hours. Statistical comparison was performed using one-way ANOVA with Tukey´s multiple comparison test. ****(p<0.0001) respect to control group. TNF-α: Tumor necrosis factor α.


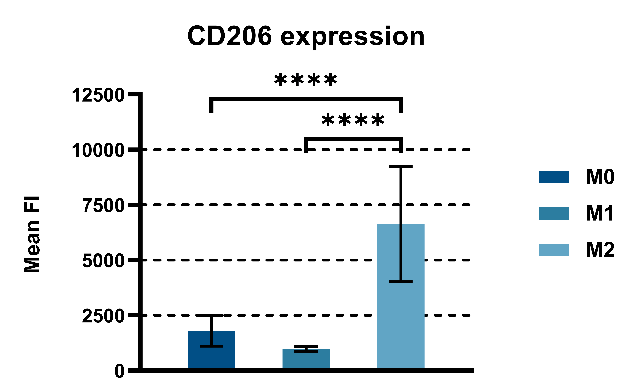


**Figure S3.** Expression of the receptor CD206 in M0, M1 and M2 macrophages. Statistical comparison was performed using one-way ANOVA with Tukey´s multiple comparison test. ****(p<0.0001). FI: fluorescence intensity.

**Figure S4.** Biodistribution of HA-Man NCs loaded with DiR and intravenously injected in the MN/MCA1 fibrosarcoma mouse model, with or without the pre-injection (10 minutes before NCs) of the liposomal liver buffering agent (Nanoprimer^™^). **A)** *In vivo* signal corresponding to the amount of NCs in the liver during the 1^st^ hour of injection is monitored by IVIS, selecting the ROI corresponding to the anatomical location of the liver. **B)** *Ex vivo* signal at the end of the experiment (24 hours) is evaluated by IVIS in excised organs (whole organs: spleen, lungs, liver and intestines).

**Synthesis of new compounds**

**2-[2-(2-Azidoethoxy)ethoxy]ethyl 2,3,4,6-tetra-*O*-acetyl-α-D-mannopyranoside (1).** 2-[2-(2-Azidoethoxy)ethoxy]ethanol (320 mg, 1.83 mmol) and 2,3,4,6-tetra-O-acetyl-α-D-mannopyranosyl trichloroacetimidate (449 mg, 0.913 mmol) were dissolved in dry CH_2_Cl_2_ (8.22 mL) under Ar and cooled to 0 °C. Dry 4 Å molecular sieves were added and the mixture was stirred at 0 °C for 20 min. Then, a solution of BF_3_·Et_2_O (11.4 μL, 0.091 mmol) in CH_2_Cl_2_ (0.913 mL) was added dropwise and the reaction was stirred at rt overnight. After addition of Et_3_N (50 μL), the reaction mixture was filtered through Celite and evaporated under reduced pressure. The crude product was purified by automated MPLC (gradient from hexane to 60% EtOAc/hexane, neutral alumina, 30 min) to give **1** as a colorless syrup (332 mg, 72%). ^1^H NMR (500 MHz, CDCl_3_) δ: 5.36 (dd, *J* = 10.0, 3.4 Hz, 1H), 5.30 (d, *J* = 10.0 Hz, 1H), 5.28 – 5.26 (m, 1H), 4.87 (d, *J* = 1.7 Hz, 1H), 4.29 (dd, *J* = 12.2, 5.0 Hz, 1H), 4.10 (dd, *J* = 12.3, 2.5 Hz, 1H), 4.06 (ddd, *J* = 9.9, 5.0, 2.4 Hz, 1H), 3.86 – 3.77 (m, 1H), 3.71 – 3.64 (m, 9H), 3.40 (t, *J* = 5.1 Hz, 2H), 2.15 (s, 3H), 2.10 (s, 3H), 2.04 (s, 3H), 1.99 (s, 3H). ^13^C NMR (75 MHz, CDCl_3_) δ: 170.5, 169.8, 169.7, 169.5, 97.5, 70.6, 70.5, 69.9, 69.4, 68.9, 68.2, 67.2, 66.0, 62.2, 50.4, 20.5. IR (neat, ATR) ν_max_: 2884, 2103, 1743, 1369, 1216, 1045 cm^-1^. HRMS (APCI-FIA-TOF, positive mode, *m/z*): 506.1997. Calcd for [M+H]^+^, C_20_H_32_N_3_O_12_: 506.1986.

^1^H NMR spectrum (500 MHz, CDCl_3_) of **1**

^13^C NMR spectrum (75 MHz, CDCl_3_) of **1**

IR spectrum of **1**

**2-[2-(2-Azidoethoxy)ethoxy]ethyl-α-D-mannopyranoside (2).** Aqueous KOH (3.75 mL, 1 M) was added to a solution of **1** (190 mg, 0.375 mmol) in EtOH (5 mL). The solution was stirred at rt for 1 h 30 min and them was neutralized with Amberlite IR-120, filtered, and evaporated. The residue was purified by automated MPLC (CH_2_Cl_2_/MeOH 20%, silica gel, 15 min) to give pure **2** (120 mg, 95%) as a colorless syrup. ^1^H NMR (500 MHz, D_2_O) δ: 4.93 (d, *J* = 1.7 Hz, 1H), 4.00 (dd, *J* = 3.5, 1.7 Hz, 1H), 3.95 – 3.89 (m, 2H), 3.86 (dt, *J* = 5.7, 3.5 Hz, 1H), 3.82 – 3.67 (m, 12H), 3.54 (t, *J* = 5.0, 2H). ^13^C NMR (75 MHz, D_2_O) δ: 100.6, 73.4, 71.1, 70.6, 70.3, 70.2, 70.1, 69.9, 67.4, 67.0, 61.6, 50.8. IR (neat, ATR) ν_max_: 3369, 2919, 2872, 2102, 1060 cm^-1^. HRMS (ESI-FIA-TOF, negative mode, *m/z*): 336.1419. Calcd for [M-H]^-^, C_12_H_23_N_3_O_12_: 336.1409.

^1^H NMR spectrum (500 MHz, D_2_O) of **2**

^13^C NMR spectrum (75 MHz, D_2_O) of **2**

IR spectrum of **2**

**2-[2-(2-Aminoethoxy)ethoxy]ethyl-α-D-mannopyranoside (3).** Pd/C (7.0 mg, 20%) was added to a solution of **2** (35 mg, 0.095 mmol) in aq HCl (0.95 mL, 2M) and MeOH (3 mL). The mixture was stirred under H_2_ (1 atm) for 6 h. Then, the catalyst was removed by filtration through Celite, and the filtrated was concentrated to give **3** as a colourless foam (32 mg, 97%). ^1^H NMR (500 MHz, D_2_O) δ: 4.93 (d, *J* = 1.7 Hz, 1H), 4.00 (dd, *J* = 3.4, 1.7 Hz, 1H), 3.95 – 3.89 (m, 2H), 3.85 (dt, *J* = 6.4, 3.5 Hz, 1H), 3.83 – 3.66 (m, 12H), 3.26 (t, *J* = 5.1 Hz, 2H). ^13^C NMR (75 MHz, D_2_O) δ: 102.8, 75.6, 73.4, 72.8, 72.5, 72.4, 69.6, 69.3, 69.2, 63.8, 42.0. IR (neat, ATR) ν_max_: 3317, 2914, 1644, 1056 cm^-1^. HRMS (ESI-FIA-TOF, positive mode, *m/z*): 312.1661. Calcd for [M+H]^+^, C_12_H_26_NO_8_: 312.1658.

^1^H NMR spectrum (500 MHz, D_2_O) of **3**

^13^C NMR spectrum (75 MHz, D_2_O) of **3**

IR spectrum of **3**

**HA-Man (4).** A solution of DMTMM (55 mg, 0.199 mmol) and **3** (7.45 mg, 0.024 mmol) in 1 mM phosphate buffer (pH 6.5, 0.4 mL) was added to a solution of **HA** (40 mg, 0.10 mmol) in 1 mM phosphate buffer (pH 6.5, 0.9 mL). The reaction mixture was stirred at 70 ºC for 2 h, and then was ultrafiltered (YM5; 4 × 30 mL sat NaHCO_3_, 3 × 30 mL H_2_O) and lyophilized to afford **HA-Man** (37.5 mg, DS 15.6%; coupling yield 65%; mass recovery 85%). ^1^H NMR (500 MHz, Dfilter 120 ms, D_2_O) δ: 4.90 (s, 0.16H), 4.54 (br s, 1H), 4.45 (br s, 1H), 4.05-3.25 (m, 12.81H), 2.02 (s, 3H).

^1^H NMR spectrum (500 MHz, Dfilter 120 ms, D_2_O) of **4**
